# Cytosponge-TFF3 Testing can Detect Precancerous Mucosal Changes of the Stomach

**DOI:** 10.1016/j.cgh.2021.07.047

**Published:** 2022-06

**Authors:** ANDREAS V. Hadjinicolaou, ALEXANDER A. Azizi, MARIA O’Donovan, IRENE Debiram, REBECCA C. Fitzgerald, MASSIMILIANO Di Pietro

**Affiliations:** MRC Cancer Unit, University of Cambridge, Cambridge, United Kingdom; Department of Digestive Diseases, Addenbrooke's Hospital, Cambridge, United Kingdom; Department of Clinical Pharmacology and Therapeutics, Addenbrooke’s Hospital, Cambridge, United Kingdom; Department of Histopathology, Addenbrooke's Hospital, Cambridge, United Kingdom; MRC Cancer Unit, University of Cambridge, Cambridge, United Kingdom; MRC Cancer Unit, University of Cambridge, Cambridge, United Kingdom; MRC Cancer Unit, University of Cambridge, Cambridge, United Kingdom; Department of Digestive Diseases, Addenbrooke's Hospital, Cambridge, United Kingdom

## Abstract

Gastric intestinal metaplasia (GIM) and gastric atrophy (GA) are associated with increased risk of gastric cancer and are indications for endoscopic surveillance when affecting the proximal stomach.^1^ Endoscopic screening is not cost-effective in areas with low-moderate incidence of gastric cancer^2^; noninvasive methods to detect GIM/GA are currently lacking.^3^

Gastric intestinal metaplasia (GIM) and gastric atrophy (GA) are associated with increased risk of gastric cancer and are indications for endoscopic surveillance when affecting the proximal stomach.^1^ Endoscopic screening is not cost-effective in areas with low-moderate incidence of gastric cancer^2^; noninvasive methods to detect GIM/GA are currently lacking.^3^

Cytosponge-TFF3 (Europlaz, Essex, UK) is a capsule on a string device coupled to a biomarker for the intestinal metaplasia (IM).^4^ In a recent randomized controlled study in primary care (BEST3 trial), Cytosponge-TFF3 led to a 10-fold increase in the diagnosis of Barrett’s esophagus (BE) among patients with reflux symptoms.^5^ We aimed to investigate whether Cytosponge-TFF3 can also detect GIM/GA.

We performed a post hoc analysis of the BEST3 trial data (registration ISRCTN68382401).^5^ Patients who received a Cytosponge-TFF3 test and an endoscopy with gastric biopsies were included. We excluded patients who had (1) endoscopic or histologic evidence of BE, (2) isolated IM at the gastroesophageal junction IM, (3) gastric biopsies exclusively from gastric cardia or fundic gland-type polyp, and (4) incomplete endoscopic or histologic data. In the BEST3 trial gastric biopsies were taken as per clinical indication apart from a single cardia biopsy, which was mandated by the protocol.^5^ The presence of *Helicobacter pylori* was assessed on histology by hematoxylin and eosin staining, with the addition of immunohistochemistry when inflammatory features were seen. The rate of GA and/or GIM diagnosis was compared in TFF3 positive (TFF3+) and TFF3 negative (TFF3-) patients by Fisher exact test with R version 4.0.3.

A total of 292 individuals received both a Cytosponge-TFF3 test and an endoscopy. After exclusion of patients with incomplete data (n = 18), histologic findings of BE or gastroesophageal junction IM (n = 134), or no gastric biopsies or fundic polyp/gastric cardia biopsies only (n = 83), 57 patients were included in the final analysis (TFF3+, n = 44; TFF3-, n = 13).^5^ In the TFF3+ and TFF3- groups, 84% and 77%, respectively, had biopsies taken from the proximal stomach (fundus or body), whereas the remaining had distal gastric biopsies only. Baseline characteristics including age, sex, and ethnicity did not differ significantly between the 2 groups.

In the study cohort, 22.8% had GIM/GA suspected at endoscopy (12 in TFF3+ group and 1 in the TFF3- group), but this was confirmed histologically in 5 cases only. *H pylori* was detected in 22.7% and 7.7% of patients in the TFF3+ and TFF3- groups, respectively (*P* = .43).

In the TFF3+ group, 34.1% of patients (n = 15) were diagnosed with GIM/GA. One of these patients was a 79-year-old man with mild reflux symptoms whose biopsies showed widespread gastric IM in the gastric body and early adenocarcinoma in the antrum, which was treated with a curative endoscopic resection. None of the patients in the TFF3- group was diagnosed with GA/GIM. In patients with no evidence of BE, TFF3 positivity was significantly associated with the presence of GA/GIM in gastric biopsies (*P* = .013) (Figure 1). Among patients with BE, 40 received gastric biopsies. Of these, only 2 (5%) showed histologic evidence of GA/GIM.

**Figure 1:**
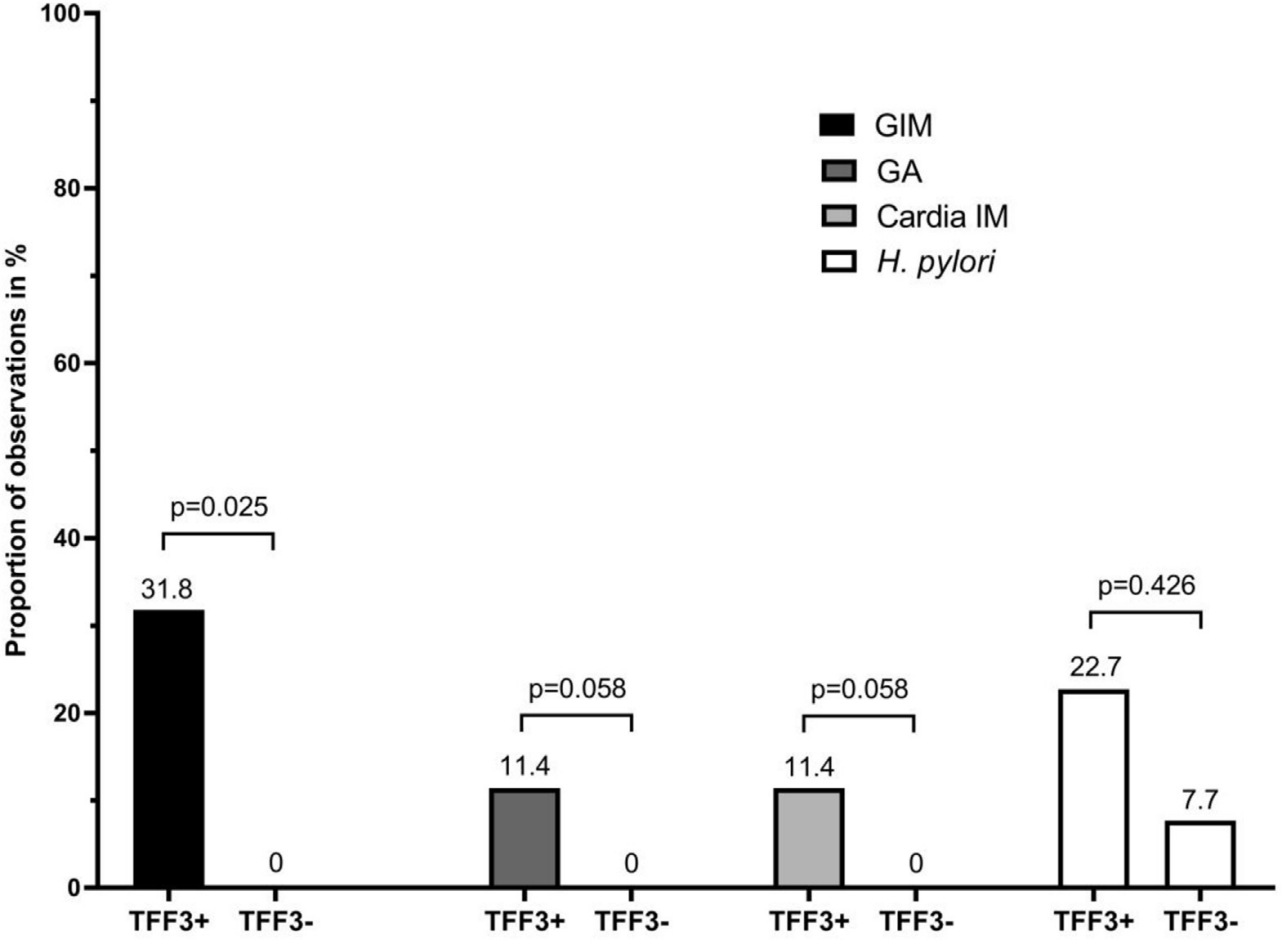
Distribution of biopsy findings in TFF3+ and TFF3- groups. Individual histologic findings are represented in the 2 groups with different color coding. The proportion of positive findings (GA, GIM, and cardia IM) does not add up to 100% because they can be diagnosed within the same patient.

Although much of the clinical research interest for Cytosponge has focused to date on esophageal pathologies, we here provide evidence that Cytosponge-TFF3 testing can detect premalignant conditions in the stomach, including GIM and GA.^5^ Although it is premature to apply these findings to a gastric cancer screening setting, they are important to be considered when performing an endoscopy in patients with positive Cytosponge-TFF3 testing. GIM/GA can have patchy distribution and be missed at standard endoscopy,^6^ particularly in patients referred for reflux, where the focus is on diagnosis of esophageal pathologies. In this cohort, less than one-third of patients with GIM/GA were suspected endoscopically.

This study has limitations. First, the population investigated in the original randomized trial is a reflux-predominant cohort. Given the inverse incidence trend of gastric and esophageal adenocarcinoma, the results of this study are not directly extendable on a screening setting for gastric cancer.^7^ Second, in the BEST3 trial most of the patients who received an endoscopy were TFF3+, therefore we expect that the sensitivity of Cytosponge-TFF3 may be lower than that detected here. Finally, there was no systematic gastric biopsy sampling, which could have led to underdiagnosis of gastric pathology. A proportion of the false-positive Cytosponge-TFF3 result is still expected to derive from focal IM at the cardia and gastroesophageal junction, which are common but have low cancer risk.^8^

In conclusion, these data indicate that patients with Cytosponge-TFF3 positive test and no endoscopic evidence of BE should receive vigilant inspection of the stomach to search for signs of GIM or GA. Prospective studies are required to confirm the utility of Cytosponge-TFF3 testing for stomach pathologies.
